# Characterization of microbial response to petroleum hydrocarbon contamination in a lacustrine ecosystem

**DOI:** 10.1007/s11356-021-13885-8

**Published:** 2021-04-19

**Authors:** Emilio D’Ugo, Milena Bruno, Arghya Mukherjee, Dhrubajyoti Chattopadhyay, Roberto Giuseppetti, Rita De Pace, Fabio Magurano

**Affiliations:** 1 Department of Infection Diseases, National Institute of Health, Viale Regina Elena 299, 00161 Rome, Italy; 2 Core Facilities, National Institute of Health, Rome, Italy; 3 Center for Genetic Engineering and the Department of Biotechnology, University of Calcutta, Calcutta, India; 4 Department of Foggia, Experimental Zooprophylactic Institute of Puglia and Basilicata Regions, Foggia, Italy

**Keywords:** Lake Pertusillo, Petroleum hydrocarbon, Aromatic hydrocarbon degradation, Lake microbiome, Oil spill, Metagenomics, Freshwater ecosystem, Bioinformatics, Metabolic reconstruction, Sustainable use of waters

## Abstract

Microbiomes of freshwater basins intended for human use remain poorly studied, with very little known about the microbial response to in situ oil spills. Lake Pertusillo is an artificial freshwater reservoir in Basilicata, Italy, and serves as the primary source of drinking water for more than one and a half million people in the region. Notably, it is located in close proximity to one of the largest oil extraction plants in Europe. The lake suffered a major oil spill in 2017, where approximately 400 tons of crude oil spilled into the lake; importantly, the pollution event provided a rare opportunity to study how the lacustrine microbiome responds to petroleum hydrocarbon contamination. Water samples were collected from Lake Pertusillo 10 months prior to and 3 months after the accident. The presence of hydrocarbons was verified and the taxonomic and functional aspects of the lake microbiome were assessed. The analysis revealed specialized successional patterns of lake microbial communities that were potentially capable of degrading complex, recalcitrant hydrocarbons, including aromatic, chloroaromatic, nitroaromatic, and sulfur containing aromatic hydrocarbons. Our findings indicated that changes in the freshwater microbial community were associated with the oil pollution event, where microbial patterns identified in the lacustrine microbiome 3 months after the oil spill were representative of its hydrocarbonoclastic potential and may serve as effective proxies for lacustrine oil pollution.

**Supplementary Information:**

The online version contains supplementary material available at 10.1007/s11356-021-13885-8.

## Introduction

Petroleum hydrocarbons are one of the most common sources of anthropogenic pollution and cause frequent contamination of both aquatic and terrestrial environments. Due to their potential adverse effects on humans and the environment, primarily manifested through their mutagenic, carcinogenic, endocrine-disrupting, and teratogenic properties, petroleum hydrocarbons represent a public health concern in routine monitoring as their presence, if undetected, may cause irreparable damage (Zhang et al. 2016; Bashir et al. 2020). Releases of petroleum hydrocarbons in the environment due to production, operational use, and transportation are expected to only increase with an ever increasing global energy demand. The development of effective technologies and restoration strategies to overcome such anthropogenic pollution are therefore of great importance, but remain challenging. While various biological systems have been used for restoration of oil-polluted habitats, microbial restoration methods are the most commonly used and often the cheapest alternative. Such remedial technologies are based on the metabolic versatility of microbes which, unlike other complex organisms, have the capability to degrade a diversity of xenobiotic compounds. Microbes, including hydrocarbonoclastic microbes, therefore play an important role in environmental protection and human health through their active role in natural recovery, detoxification, and bioremediation (Xu et al. 2018). Recent advances in our understanding of microbial bioremediation have indicated that such processes do not occur in isolation in the environment and generally involve a consortia of microbes (Mukherjee et al. 2017). The availability of massively parallel sequencing technologies and sophisticated bioinformatic tools have facilitated holistic studies on microbial bioremediation and highlighted the need to study microbiomes in their totality.

**Fig. 1:**
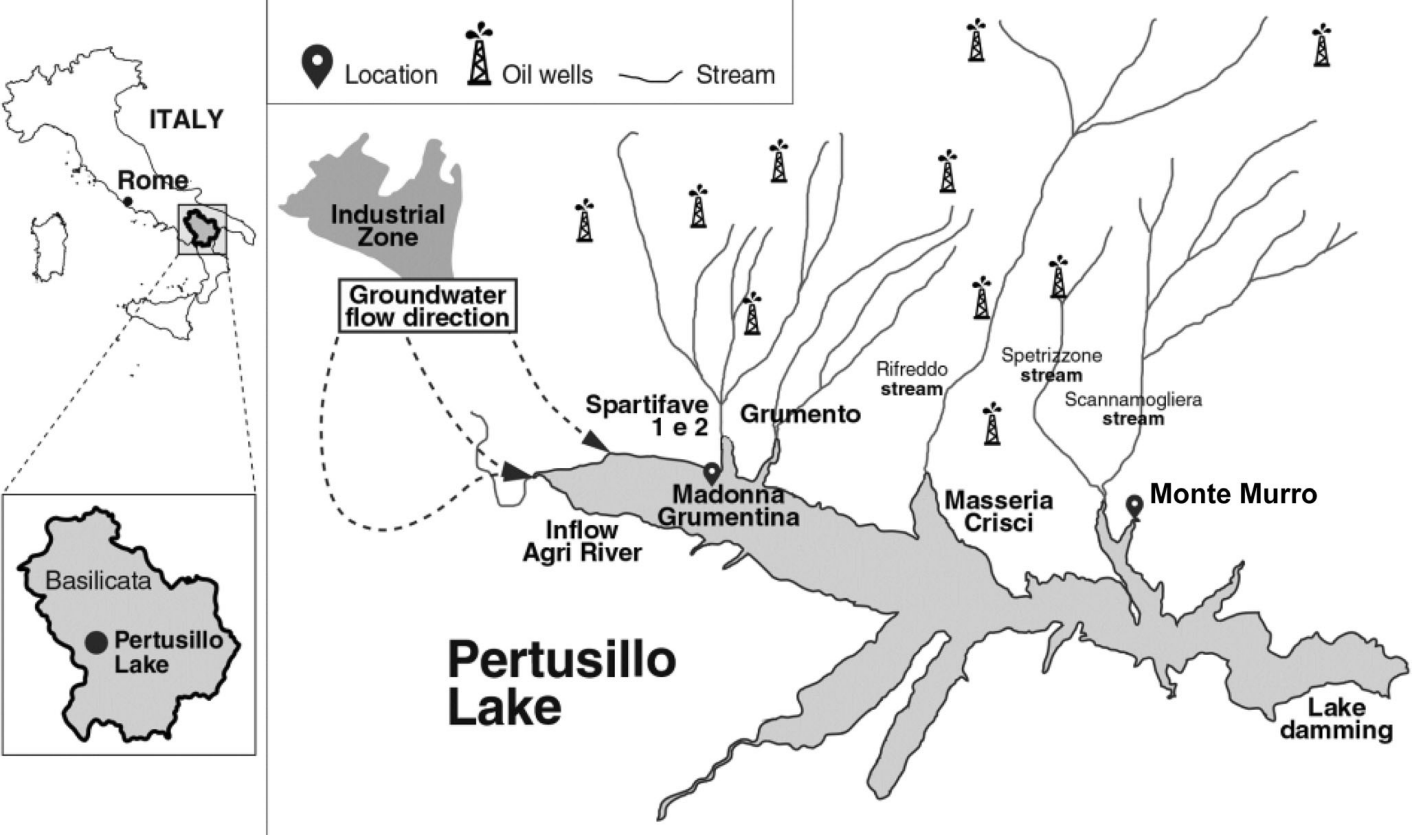
Lake Pertusillo and its surrounding area. Map of the Val d’Agri region of Italy with sites of interest including Lake Pertusillo, major oil extraction installations, groundwater flows, and streams draining into the lake, shown. The sampling sites for the present study, i.e., Madonna Grumentina and Monte Murro, are depicted with black location pins on the map

The most frequently documented contamination of water bodies in the 20th century are oil spills, with most occurring in the marine environment and being related to transport or production of crude oil (LJuhasz and Naidu 2000). Oil spills in freshwater ecosystems such as rivers and lakes, by comparison, are much rarer. The water basin and groundwater of Lake Pertusillo, an artificial freshwater reservoir in the Val d’Agri region of Italy, have been reported to be affected by oil extraction activities carried out in 27 oil wells around the lake by Ente Nazionale Idrocarburi (ENI 2019) (Fig. 1). Due to the close proximity of oil extraction activities with respect to Lake Pertusillo, the site was classified by the Italian Ministry of Environment as an environmentally sensitive site with potential for major accidents and adverse environmental impact (Ministry of the Environment 2018). Since 2010, several studies and field analyses have detected anomalous levels of heavy metal and hydrocarbons being present in the lake sediments and water (Colella 2012; Colella and D’Orsogna 2014); among others, seismicity induced by hydraulic fracturing and other injection-based oil extraction techniques have been implicated in such contaminations (Improta et al. 2015; Buttinelli et al. 2016). Furthermore, large-scale and repeated seasonal fish deaths have been observed in the lake during the last decade (De Pace et al. 2019). Recent studies carried out by the Italian Ministry of Health on the ichthyic fauna in Lake Pertusillo detected cyanotoxins, heavy metals, polychlorobiphenyles, and hydrocarbon contamination in several fish species sampled in the lake (De Pace 2015). In February 2017, Lake Pertusillo suffered a major oil spill where over 400 tons of crude oil was spilled from a nearby oil extraction plant into the lake (European Parliament 2017); although unfortunate, the oil spill offered a rare opportunity to study in situ microbial response to oil pollution in a lacustrine environment.

The coexistence of water resources in the proximity of highly polluting anthropogenic activities, such as oil extraction plants, petrochemical plants, and oil platforms represents a serious concern in conservation of freshwater ecosystems and as an extension, protection of human health (European Environment Agency 2019). However, since oil spills in freshwater environments are relatively rare, little is known of the microbial response to petroleum hydrocarbon contamination in these environments. In the present study, we have carried out culture-independent analyses of microbial communities in a lacustrine ecosystem impacted by oil spills where we have inferred changes in the lake microbiome in the aftermath of the pollution event along with metagenome prediction and consequent deductions on hydrocarbonoclastic functionalities. Our studies not only demonstrate changes in the lake microbial community due to oil pollution, but also identify principal hydrocarbonoclastic actors and major functional pathways involved in hydrocarbon degradation. Additionally, we also infer the microbial community succession in Lake Pertusillo after the oil pollution event. To our knowledge, our study represents one of the few studies carried out on oil pollution and subsequent microbial response in lacustrine ecosystems, and insights gained from the study should be useful in better monitoring and sustainable use of freshwater resources as well as designing of novel bioremediation strategies.

## Materials and methods

### Study site and sample collection

Samples were collected before and after the pollution event in February 2017, where 400 tons of crude oil was spilled from a nearby oil extraction plant run by Ente Nazionale Idrocarburi into Lake Pertusillo. Details for collected samples are given in Table 1. Certified analyses for detection of total petroleum hydrocarbons (TPH) in water samples were performed by the Regional Agency for Environment Protection of Basilicata (ARPAB) and civic associations (ARPAB 2017a; Cova Contro 2017a, b). Samples 1B, M16, and MM represent superficial samples and were collected from just under the water surface, whereas sample 2B was collected near the bottom of the lake; such a sampling design was pursued in order to understand changes in the microbial community structure along the water column. Water samples were collected using 1-L Pyrex glass bottles and stored in ice (4 °C) boxes; samples were later processed using a previously described ultrafiltration protocol (D’Ugo et al. 2016). The elutriate (50 mL) was used for downstream laboratory investigations. To study the metabolic potential of the microbiome, microcosms were prepared using sample 1B as an inoculum; the microcosm (sample 1B_LB) was supplemented with Luria-Bertani (LB) broth and incubated at 30 °C for a week. Inoculum from 1B_LB was further employed to enrich hydrocarbonoclastic bacteria in a microcosm supplemented with diesel (D) as the sole carbon source, i.e., sample 1B_LB_D, and incubated at 30 °C for a week.

**Table 1:** Description of sampling sites at Lake Pertusillo

Sample index	Collection site	Coordinates	Sampling date	Total petroleum hydrocarbons (at time of collection)	Sequence read archive (SRA) accession number
1B	Madonna Grumentina	N 40.29172 E 15.92957	May 22, 2017 (after oil spill)	900 μg/L	SRX3362329
2B	Madonna Grumentina	N 40.29172 E 15.92957	May 22, 2017 (after oil spill)	900 μg/L	SRX3362330
M16	Madonna Grumentina	N 40.29172 E 15.92957	Jan. 15, 2016 (before oil spill)	Not detected	SRX3362327
MM	Monte Murro	N 40.28885 E 15.97414	May 12, 2015 (before oil spill)	Not detected	SRX3362325
1B_LB	1B derived laboratory sample	Not applicable	Not applicable	Not analyzed	SRX3362328
1B_LB_D	1B derived laboratory sample	Not applicable	Not applicable	Diesel used as sole energy source	SRR6255871

For extraction of total genomic DNA, 5 mL of environmental and microcosm samples were centrifuged at 6000 rcf for 10 min at room temperature. The pellet was used for DNA extraction. Total environmental DNA was extracted from samples using the Genomic DNA tissue kit (Machery-Nagel, Thermo Fisher, Italy) and quantified using a spectrophotometer (NanoDrop, Thermo Fisher). High-throughput sequencing of V3-V4 and V3-V5 regions of the 16S rRNA gene for the microbiome was performed by Eurofins MWG Operon (Ebersberg, Germany). All sequencing data generated in the study has been deposited in the NCBI Sequence Read Archive (SRA) under the BioProject number PRJNA412797.

### Bioinformatic analysis

16S rRNA sequences generated in the study were evaluated for quality using FastQC (Andrews 2010), and subsequently processed in mothur (Schloss et al. 2009) with trimming of adapters, primers, and barcodes. Additional quality control was performed using the following criteria: maxhomop = 6, maxambig = 0, minlength = 200, qwindowaverage = 30, bdiffs = 1, pdiffs = 2, and tdiffs = 2. Mothur was subsequently used to align quality-filtered sequences to the SILVA (Quast et al. 2013) database. Chimeric sequences were removed using the mothur implementation of vsearch (Rognes et al. 2016). QIIME (Quantitative Insights Into Microbial Ecology) was used for open reference OTU (Operational Taxonomic Units) calling and taxonomic assignment of high-quality 16S rRNA sequences against the SILVA nr v132 release. Taxonomic classification was summarized in QIIME and subsequently visualized in R with ggplot2 (Wickham 2009). Metabolic reconstruction for 16S rRNA datasets was conducted in PICRUSt v2.0.0 (Douglas et al. 2019). Metagenomes for the 16S rRNA datasets were predicted using BIOM abundance files and representative sequence files as input; these were generated in QIIME during OTU calling and taxonomic assignment, respectively. Predicted metagenomes were further collapsed into MetaCyc pathways by the MinPath (Langille et al. 2013) implemented within PICRUSt 2.0.0 and abundances for the same were also obtained. Inferred metagenomes were visualized as cladograms generated with the standalone graphical tool GraPhlan v0.95 (Ye and Doak 2009).

## Results

### Changes in microbial community composition observed at the Phylum level

To better understand the changes in hydrocarbonoclastic properties of the Lake Pertusillo microbiome after the oil spill, bioinformatic analysis was directed towards the study of bacteria putatively capable of degrading hydrocarbons. To this end, 16S rRNA gene sequence analysis of lake samples was performed for samples collected both before (MM and M16) and after oil spill (1B and 2B) (Fig. 1). Comprehensive taxonomic analysis was carried out for each sample, along with predictive metabolic reconstruction to elucidate the metabolic potential of the microbial community.

Taxonomic analysis of Lake Pertusillo samples revealed that phylum Proteobacteria was the predominant phylum in oil-contaminated samples (1B and 2B). The proportion of phylum Proteobacteria in the microbiome increased notably from 52% before oil spill (sample M16), to 81% and 77% (samples 1B and 2B, respectively) after the pollution event (Supplementary Figure S1). Additionally, the M16 sample, collected at same coordinates as samples 1B and 2B before the oil spill, showed much higher levels (24%) of bacteria assigned to phylum Actinobacteria (Supplementary Figure S1). The phylum was appreciably reduced in proportion in samples 1B and 2B (5.4% and 4.3%, respectively) compared to the M16 sample indicating an adverse response of actinobacterial microbes to oil contamination in the lake. Surprisingly, in the MM sample, Actinobacteria represented the dominant phylum and not Proteobacteria (Supplementary Figure S1).

To understand if hydrocarbonoclastic properties of Lake Pertusillo microbes were primarily sequestered in the Proteobacterial phylum, 1B samples were enriched for hydrocarbon-degrading bacteria. To do this, 1B samples were added to a rich medium (Luria-Bertani (LB) broth) without any hydrocarbons and incubated for a week (sample 1B_LB). Taxonomic analysis of the 1B_LB microbiome revealed that in the absence of hydrocarbons, the microbial community composition changes considerably; this was represented by a decline of phylum Proteobacteria (from 81% to 23%) and increase of phylum Firmicutes (from 0.1% to 75%; Supplementary Figure S1 and S2). When hydrocarbons were re-introduced into the 1B_LB sample (1B_LB_D), where diesel (D) was provided as the sole energy source, the microbiota displayed a rapid shift towards a Proteobacteria-dominated population (99%; Supplementary Figure S2). Thus, it can be observed that, while environmental samples 1B and 2B (total petroleum hydrocarbon, TPH: 900 μg/L) and the microcosm with diesel (1B_LB_D) maintain a microbiome structure dominated by Proteobacteria, an absence of hydrocarbons in the 1B_LB sample caused a drastic reduction of Proteobacteria and increase in Firmicutes; taken together, these indicate that most hydrocarbonoclastic bacteria in Lake Pertusillo belong to phylum Proteobacteria.

### Changes in microbial community composition observed at the Genus level

To understand the Lake Pertusillo microbiome further and how it changed when exposed to petroleum hydrocarbon contamination, we analysed the Pertusillo microbiome before and after the oil spill at the genus level and compared it with microbiomes from two pristine Bulgarian reservoirs (Supplementary Table S1) (Iliev et al. 2017). Our analyses revealed that contaminated samples from Lake Pertusillo were enriched for alphaproteobacterial and betaproteobacterial genera with specialized hydrocarbonoclastic competence, as has been observed previously in other oil-contaminated ecosystems (Table S1) (Yang et al. 2014; Kostka et al. 2011). *Hydrogenophaga* sp., a member of the Betaproteobacteria family Comamonadaceae and previously reported in oil sludge samples in Saudi Arabia (Albokari et al. 2015), was found to be notably enriched in the two contaminated samples 1B and 2B (1B: 22.7%; 2B: 8.9%) in comparison to lake samples before oil spills (MM: 0.0%, M16: 0.0%) (Supplementary Table S1). Similar differences in abundances were observed for *Hydrogenophaga* when compared to pristine Bulgarian lacustrine ecosystems where all five sites analyzed showed a *Hydrogenophaga* abundance under 1% (Supplementary Table S1).

Another member of class Betaproteobacteria, *Acidovorax* sp., is also well represented in the oil-contaminated lake microbiome, showing a notable increase compared to unpolluted Lake Pertusillo samples (1B: 3.5%; 2B: 12.2% ; MM 0.0% and M16 0.0%) and pristine Bulgarian lake samples (Supplementary Table S1).

*Afipia* sp., belonging to Class Alphaproteobacteria, was enriched in the two contaminated Lake Pertusillo samples (1B: 3.5%; 2B: 12.2%) compared to all other lake samples (Supplementary Table S1). Interestingly, *Reyranella* sp. (Class Alphaproteobacteria) was also highly enriched in the oil-contaminated Lake Pertusillo samples (1B: 7.3%; 2B: 2.%) in comparison to unpolluted Lake Pertusillo samples and Bulgarian water basin samples (Supplementary Table S1).

*Dyadobacter* sp., a member of Phylum Bacteroidetes, was found to be notably enriched only in the oil-contaminated sample 1B (1B: 2.79%, Supplementary Table S1). The versatile genus *Variovorax* (Class Betaproteobacteria) was also enriched only in sample 1B (1B: 17.8 %; Supplementary Table S1).

In samples analyzed along the contaminated water column, i.e., samples 1B and 2B, we detected two Alphaproteobacterial genera, *Sphingopyxis* (1B: 1.1%; 2B: 2.2% and *Hirschia* (1B: 0.8%; 2B: 1.2% Supplementary Table S1).

### Metabolic reconstruction of Lake Pertusillo microbiomes

**Fig. 2:**
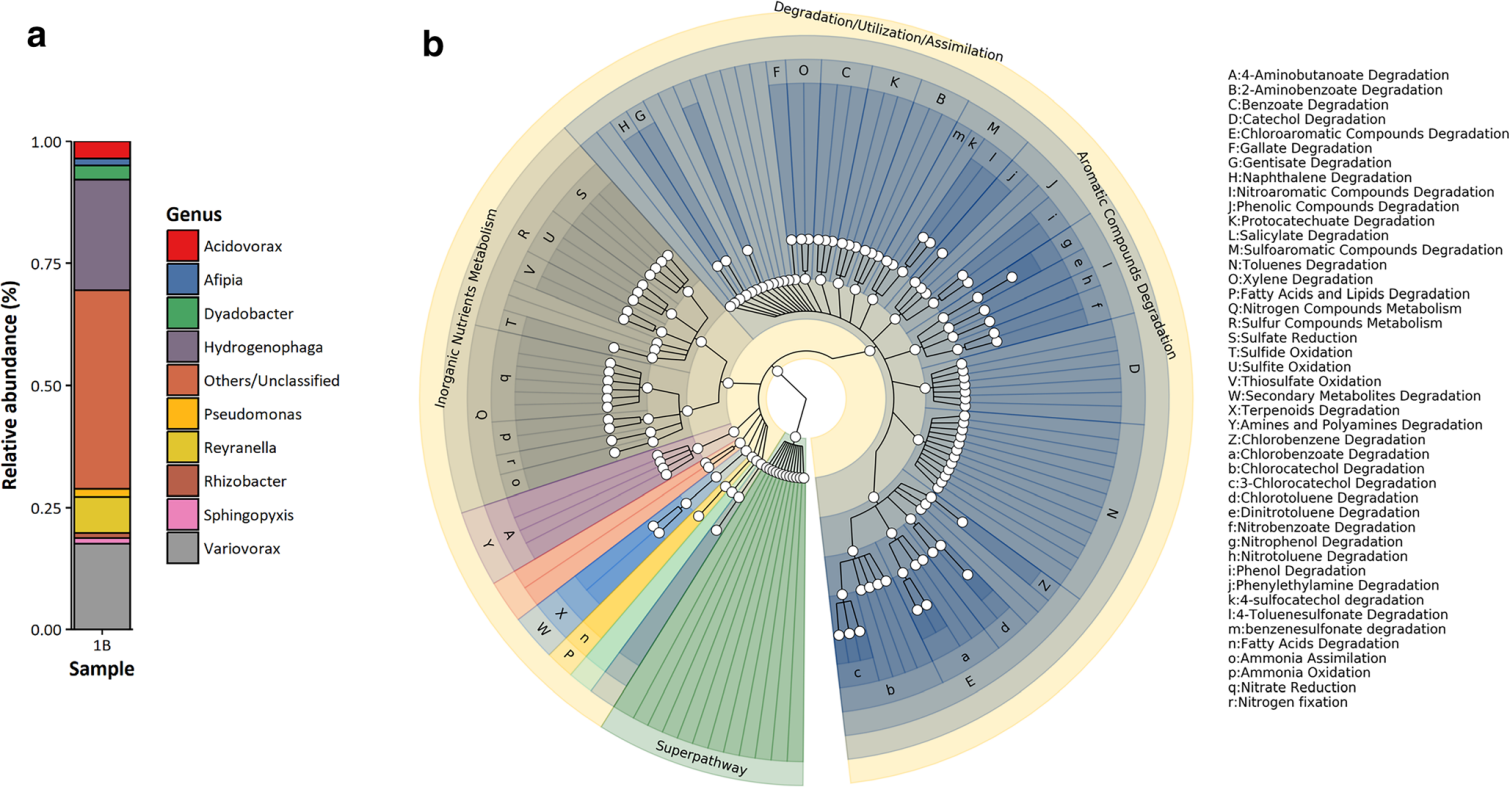
**a** Microbiome contributions of OTUs at the genus level in sample 1B. **b** Metabolic reconstruction of sample 1B and 2B. A subset of pathways related to hydrocarbon degradation and nitrogen and sulfur metabolism for samples 1B and 2B is depicted in the circular cladogram. Each ring of the cladogram from the center denotes a level of the pathway ontology. Corresponding labels are found from the outermost ring to the final clade in the sector. Each clade is depicted by a circular marker

To functionally characterize the Lake Pertusillo microbiome, metabolic reconstruction was conducted in PICRUSt v2.0.0 with metabolic pathways being inferred using MinPath. Bioinformatic analysis confirmed a hydrocarbonoclastic metabolic landscape for samples 1B and 2B, wherein multiple metabolic pathways involved in degradation of aromatic hydrocarbons were detected (Fig. 2). These include metabolic pathways involved in the degradation of aromatic hydrocarbons such as catechol, phenol, phthalate, ethylbenzene, protocatechuate, and toluene; metabolic pathways involved in mineralization of chlorinated hydrocarbons such as PCBs and other chloroaromatic and sulfonated aromatic hydrocarbons were also detected (Fig. 2b and Supplementary Table S2).

## Discussion

Microbial response to oil contamination in freshwater ecosystems, especially lacustrine habitats, is poorly studied. Studies on the microbial community composition and metabolic landscape of polluted lacustrine environments contribute to our understanding of the dynamics of microbial response and in turn, facilitate better conservation of water resources. Recently, high-throughput sequencing has facilitated the advancement of microbial ecological studies in polluted habitats with bioinformatic approaches being used to reveal metagenomic characteristics of bacterial response to petroleum hydrocarbon contamination in diverse ecosystems. Using 16S rRNA gene sequences as an input in PICRUSt (Douglas et al. 2019) and other similar bioinformatic tools (Aßhauer et al. 2015), it is possible to predict metagenomics features and functional compositions of microbiomes; subsequently, critically important and enriched taxa and functional pathways can be inferred as biomarkers that can be effectively used to distinguish diverse oil-polluted environments (Mukherjee et al. 2017).

### Proteobacteria dominate the oil-polluted lacustrine microbiome

In the present study, Proteobacteria were found to be the dominant phylum in the oil polluted Lake Pertusillo microbiome. The Proteobacterial dominance of an oil-contaminated Lake Pertusillo microbiome is in agreement with previous observations from other ecosystems which suffered oil pollution events (Mukherjee et al. 2017). For example, Yang et al. have previously reported a similar shift in microbial community structure towards phylum Proteobacteria upon oil contamination in permafrost habitats (Yang et al. 2014). Members of this phylum, such as those belonging to the classes Alphaproteobacteria, Betaproteobacteria, and Gammaproteobacteria, are known to be capable of using various hydrocarbons as their sole carbon source (Kostka et al. 2011). Unsurprisingly, when evaluated at the genus level, contaminated samples from Lake Pertusillo were found to be enriched for alphaproteobacterial and betaproteobacterial genera.

*Hydrogenophaga* sp., a member of the Betaproteobacteria family Comamonadaceae, was found to be notably enriched in the two contaminated samples 1B and 2B. The genus has previously been isolated from microbial consortia in benzene-contaminated sites (Fahy et al. 2008) along with *Acidovorax* sp. and *Pseudomonas* sp., both of which were also detected in our study. The presence of *Hydrogenophaga* sp. was also described in microcosms set up using groundwater from a BTEX (benzene, toluene, ethylbenzene, and xylenes) contaminated site as inoculum with a mixture of toluene and benzene used as sole carbon sources (Aburto and Peimbert 2011). *Hydrogenophaga* sp. have additionally been reported to play an important role in PAH-degrading (polycyclic aromatic hydrocarbons) microbial communities (Martin et al. 2012). Furthermore, a role for *Hydrogenophaga* in polychlorinated biphenyls (PCBs) degradation has been proposed previously (Lambo and Patel 2006). These data are largely in agreement with a previous study, which detected PCBs in ichthyic fauna of Lake Pertusillo (De Pace 2015) and with the ARPAB (ARPAB 2017b) that stresses that PCBs such as PAH are present in the sediment of the entire reservoir with higher concentrations in 2017. Acidovorax, another betaproteobacterial genera, was well represented in the oil-contaminated samples. *Acidovorax* has previously been found to be the most abundant dominant bacterial species in the sludge of an Alberta oil sand tailing pond (Singleton et al. 2009). Like other members of Comamonadaceae, *Acidovorax* is frequently encountered in association with PAH degradation (Singleton et al. 2018). Denitrifying *Acidovorax* sp. has been isolated from terrestrial subsurface sediments exposed to mixed-waste contamination; in particular, this genus has been detected in nitrate-reducing microbial consortia cultivated with alkylated aromatic compounds, revealing their important role in hydrocarbons and nitrate-polluted waters (Sperfeld et al. 2018). The versatile betaproteobacterial genus *Variovorax* sp. was enriched only in sample 1B; the genus has been previously reported for hydrocarbonoclastic properties such as biosynthesis of biosurfactants (Franzetti et al. 2012) and PAH degradation in the presence of nitrates (Eriksson et al. 2003).

*Afipia* sp., belonging to class Alphaproteobacteria, was enriched in the two contaminated Lake Pertusillo samples. Similar to *Acidovorax* sp., *Afipia* sp. is a well-known degrader of polycyclic aromatic substances (Willumsen et al. 2005) and can degrade PAHs using nitrates as oxidizing agents; such combination of PAH degradation with denitrification by *Afipia* sp. has been observed in nitrate-contaminated groundwater (Green et al. 2010). *Reyranella sp.*, an alphaproteobacterial genus which was found to be enriched in oil-contaminated samples, has been previously reported in degradation of asphaltenes, a particularly recalcitrant fraction of crude oil (Song et al. 2018). In samples analyzed along the water column, i.e., samples 1B and 2B, the alphaproteobacterial genera *Sphingopyxis* and *Hirschia* were detected. Both these genera have been previously identified to play important roles in microbiome successions in relation to oil spills (Rodriguez et al. 2015). In the first phase (during the oil spill), sand microbiomes in beaches affected by the Deep Water Horizon oil spill showed a prevalence of microbes that degrade aliphatic hydrocarbons and were replaced in the second phase (2–3 months after the oil spill) with a PAH-degrading microbial community enriched in *Sphingopyxis* and *Hirschia* (Rodriguez et al. 2015).

*Dyadobacter* sp., a member of Phylum Bacteroidetes, was found to be enriched only in the oil-contaminated sample 1B; members of the genus are particularly specialized in degradation of complex aromatic hydrocarbons such as azaarenes, which are relatively water soluble, nitrogen-containing heterocyclic aromatic hydrocarbons that have been reported to leach from contaminated soils and sediments into aqueous habitats (Pereria et al. 1983). The isolation and study of these enriched species identified herein, among others, could help us better understand lacustrine microbial response to oil pollution and may serve as excellent tools for future bioremediation interventions and as microbial proxies for oil pollution in lakes; taken together, these will contribute to protection of freshwater ecosystems and reduction of public health risks. Indeed, recent further studies concentrated on the hydrocarbonoclastic biofilms formed on the water surface after the Lake Pertusillo oil pollution event have revealed unique electrogenic structural and biochemical properties of the biofilm and sheds further light on possible mechanisms of microbial detoxification of oil-contaminated lacustrine habitats (D’Ugo et al. 2021). Overall, the presence of a hydrocarbonoclastic microbial community characterized by an abundance of proteobacterial genera such as *Hydrogenophaga*, *Acidovorax*, *Reyranella*, and *Variovorax* among others, highlighted the lake's pollution status, indicating a community shaped by residual recalcitrant hydrocarbons derived from the oil spill 3 months earlier and with significant complex hydrocarbon degradation potential.

### Metabolic landscape of polluted lacustrine microbiome reveals microbial hydrocarbonoclastic potential

Detection of hydrocarbonoclastic pathways involved in the degradation of complex aromatic hydrocarbons in metagenomes predicted through PICRUSt is consistent with the hydrocarbon degradation potential of several genera found to be enriched in petroleum hydrocarbon-contaminated Pertusillo samples. Indeed, microbial genera such *Hydrogenophaga*, *Acidovorax*, *Reyranell*a*, Variovorax*, and others, found to be enriched in polluted samples as described above, exhibit multiple metabolic pathways involved in degradation of aromatic hydrocarbons. For example, mineralization pathways for pyrene and benzo-[a]-pyrene along with other high-molecular weight PAHs have been reported previously for *Hydrogenophaga* sp. (Yan et al. 2017). Additionally, metabolic pathways for the degradation of chloroaromatics have also been identified in *Hydrogenophaga* sp., including transformation of 2,4′-dichlorobiphenyl (2,4′-DCB) into 2- and 4-chlorobenzoic acid (2- and 4-CBA; Lambo and Patel 2006). Furthermore, degradation of sulfo-nitroaromatic compounds have also been reported for *Hydrogenophaga* sp.; indeed, Gan et al. reported the mineralization of 4-aminobenzenesulfonate (4-ABS) by *Hydrogenophaga* sp. strain PBC involving enzymatic conversions catalyzed by 4-sulfocatechol 1,2-dioxygenase, 3-sulfomuconate cycloisomerase, and 3,4-dioxygenase enzymes (Gan et al. 2011). The versatility of *Hydrogenophaga* sp. as a degrader of PAHs could explain the increased abundance of the genus in oil-contaminated samples, as mentioned above (Supplementary Table S1). PAH degrading capabilities have also been reported for other genera found to be abundant in oil-contaminated samples, i.e., *Acidovorax*, *Reyranella*, and *Variovorax*, with metabolic pathways employing diverse hydrocarbonoclastic enzymes such as ring-hydroxylating dioxygenase (Singleton et al. 2009), catechol 1,2-dioxygenase (El Azhari et al. 2010), and hydrolytic esterases (Wang and Gu 2006), among others, being identified in them.

Although it was not possible to analyze the microbial community structure of Lake Pertusillo during the oil spill, our results indicate that hydrocarbonoclastic capacities of the lacustrine microbiome in the aftermath of the oil spill were highly skewed towards mineralization of complex hydrocarbons including PAHs, choroaromatics, nitroaromatics, and sulfonated aromatic compounds with few metabolic pathways for degradation of aliphatic petroleum hydrocarbons detected. This suggests that there may have been a shift in the microbiome towards a specific hydrocarbonoclastic competence at this stage, i.e., 3 months after the oil spill, where the structure of the Pertusillo microbiome could be shaped by the residual presence of more recalcitrant, complex hydrocarbons such as benzene derivatives, toluene, PAH, PCB, and BTEX, among others, after most aliphatic hydrocarbons had been degraded. Such an observation is substantiated further by detection of several genera of microbes known to specialize in PAH and complex hydrocarbon degradation, as described above, as well as previously reported similar observations from the Deep Water Horizon oil spill where the second stage of microbial successions was dominated by PAH degrading microbes (Rodriguez et al. 2015; Martirani-Von Abercron et al. 2017). Importantly, predictive metabolic reconstruction using PICRUSt demonstrated that advanced bioinformatic pipelines can be successfully employed in dissecting complex freshwater ecosystem metabolomes, where hydrocarbonoclastic contributions of members of microbial communities in polluted habitats can be inferred and in turn, provide important insights.

## Conclusions

While multiple studies have been conducted on microbial response to petroleum hydrocarbon contamination in the marine environment, little is known about microbial response to oil spills in lacustrine and freshwater environments owing to scarcity of studies on the same. In the present study, we have described the microbial response to an oil spill in Lake Pertusillo in February 2017, including taxonomic and functional characterizations for polluted and non-polluted samples. Our study has identified several taxa such as *Hydrogenophaga*, *Variovorax*, *Reyranella*, *Acidovorax*, and *Afipia*, among others, that may be used as microbial proxies for oil pollution in freshwater environments. Additionally, functional characterization of microbial response using bioinformatic approaches identified several metabolic pathways that may be involved in mineralization of Lake Pertusillo oil pollutants. Microbial successions in an oil-contaminated Lake Pertusillo revealed similarities with observations reported from marine oil spills, where mature microbial communities specialized in degradation of recalcitrant complex petroleum hydrocarbons. The detection of a specifically modified microbial community after a suspected pollution event, in this case concerning petroleum hydrocarbons, allows us to devise hypotheses about the causes and beginning of such events, acting as a “cold case” detection even after 3 months had elapsed following the occurrence. Since most freshwater ecosystems are used as sources of potable drinking water, microbial proxies identified herein and methods for detecting alterations in the lacustrine microbiome and its metabolic potential can be valuable tools in detecting pollution events and preventing major public health complications. To our knowledge, the present study is one of the few conducted on oil spills in lacustrine environments and will contribute further to our understanding of the origins and temporal dynamics of microbiome alterations in freshwater ecosystems impacted by petroleum hydrocarbon contamination.

## Supplementary information

ESM 1 (PDF 1993 kb)
