# Supplementary material for: Characterization of microbial response to petroleum hydrocarbon contamination in a lacustrine ecosystem
Source: Environ Sci Pollut Res Int. 2021 Apr 19;28(20):26187–96. doi: 10.1007/s11356-021-13885-8 (PMC8154760; doi:10.1007/s11356-021-13885-8)
Supplement: Supplementary file 1 — (PDF 1993 kb) [file 11356_2021_13885_MOESM1_ESM.pdf]

Characterization of Microbial Response to Petroleum Hydrocarbon Contamination in a Lacustrine Ecosystem

Supplementary Information

Emilio D'Ugo[1*], Milena Bruno[2], Arghya Mukherjee[3] Dhrubajyoti Chattopadhyay[4], Roberto Giuseppetti[1], Rita De Pace[5] and Fabio Magurano[1].

[1] Department of Infection Diseases, National Institute of Health, Rome, Italy

[2] Core Facilities, National Institute of Health, Rome

[3] Center for Genetic Engineering and the Department of Biotechnology, University of Calcutta - Calcutta, India

[4] Department of Foggia, Experimental Zooprophylactic Institute of Puglia and Basilicata Regions - Foggia, Italy

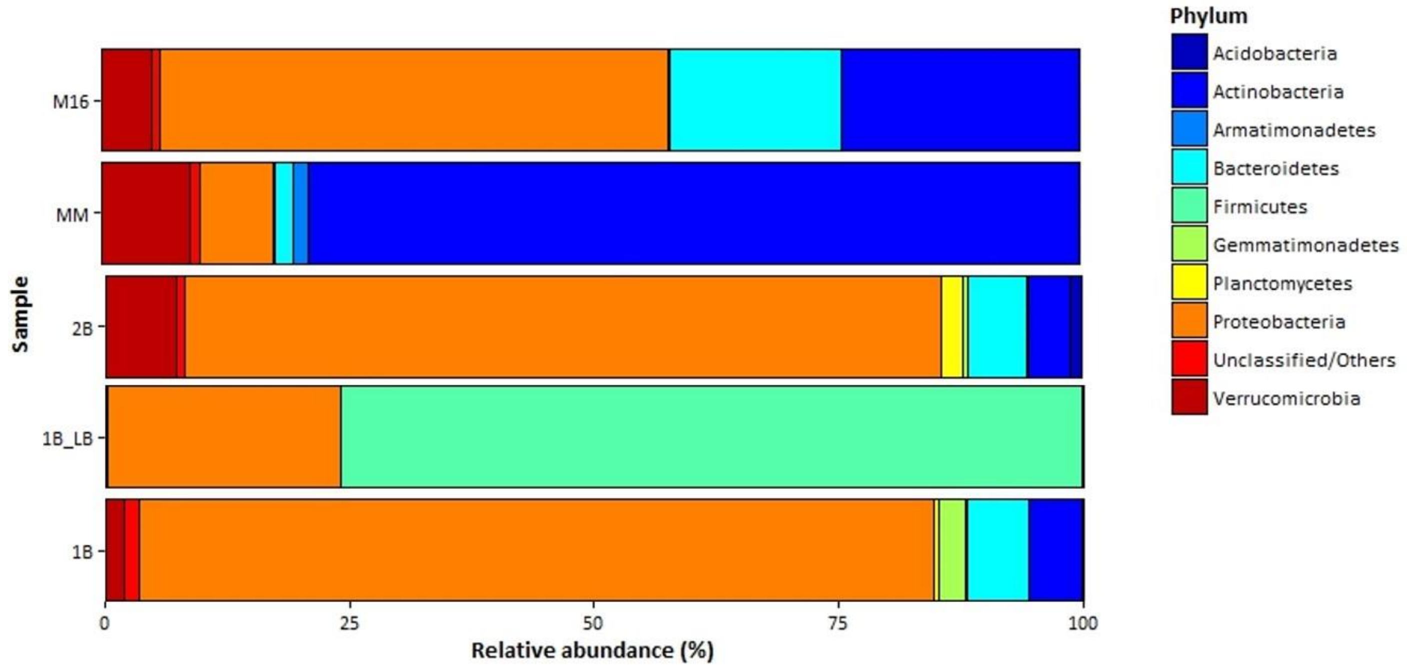

Figure S1. Phylum-level taxonomic composition of Lake Pertusillo microbiomes.

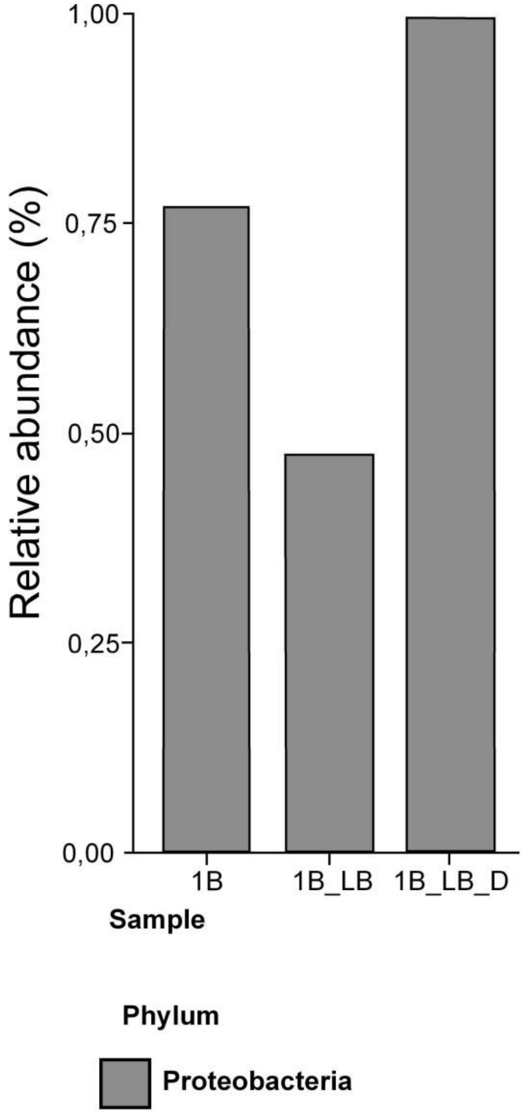

Figure S2. Phylum dynamics of Proteobacteria in the sample 1B for exposure to LB culture medium (Lauria Broth, sample 1B_LB) and for exposure of the sample 1B_LB to diesel oil as a sole energy source (sample 1B_LB_D).

Table S1. Genus level taxonomic composition of petroleum hydrocarbon contaminated (1B and 2B) and uncontaminated Lake Pertusillo samples (M16, MM) along with pristine Bulgarian reservoir samples (B1, B2, B3, CK1, CK2). [Details for Bulgarian datasets: Bioproject PRJNA352655; B3 accession number (SRX23311144), B1 (SRX2331141), CK2 (SRX2331140), CK1(SRX233142) and B2(SRX2331143)].

Genus	1B	1B_LB	2B	MM	M16	B3	B1	CK2	CK1	B2
CL500-29 marine group	0,0010728	0,0000000	0,0035569	0,1320149	0,0281645	0,0180951	0,0344646	0,0221410	0,0300625	0,0527098
Mycobacterium	0,0007450	0,0000000	0,0028794	0,0234431	0,0000000	0,0002909	0,0007917	0,0003178	0,0007170	0,0005839
hgcI clade	0,0054534	0,0000000	0,0106707	0,4761967	0,1708910	0,0354338	0,0663430	0,0599608	0,0973062	0,1198047
Aeromicrobium	0,0001192	0,0000000	0,0008469	0,0017681	0,0000000	0,0000000	0,0000000	0,0000000	0,0000000	0,0000000
Ferruginibacter	0,0001490	0,0000000	0,0182927	0,0000000	0,0272694	0,0000000	0,0010028	0,0002648	0,0001024	0,0010085
Sediminibacterium	0,0000596	0,0000000	0,0000000	0,0000000	0,0656980	0,0025601	0,0052779	0,0102230	0,0114719	0,0070598
Dyadobacter	0,0279822	0,0004293	0,0000000	0,0001310	0,0000000	0,0000000	0,0000000	0,0000000	0,0000000	0,0000000
uncultured Spirosomaceae	0,0407665	0,0001561	0,0015244	0,0000000	0,0000000	0,0000000	0,0000000	0,0000000	0,0000000	0,0000000
Fluviicola	0,0006556	0,0000000	0,0000000	0,0000000	0,0237465	0,0001746	0,0031139	0,0014831	0,0030728	0,0049896
Flavobacterium	0,0002086	0,0000000	0,0167683	0,0254076	0,0287929	0,0035492	0,0081279	0,0386673	0,0651951	0,0021233
CL500-3	0,0017284	0,0000000	0,0128726	0,0004584	0,0000000	0,0000000	0,0000000	0,0000000	0,0000000	0,0000000
uncultured Acetobacteraceae	0,0065858	0,0000000	0,0181233	0,0001310	0,0000000	0,0008146	0,0065974	0,0000000	0,0003073	0,0039280
Brevundimonas	0,0016390	0,0000000	0,0032182	0,0000000	0,0000000	0,0000000	0,0000000	0,0002119	0,0000512	0,0002123
Hirschia	0,0087612	0,0000000	0,0128726	0,0000000	0,0000000	0,0000582	0,0000000	0,0000000	0,0000000	0,0005308
Reyranella	0,0739041	0,0000000	0,0277778	0,0000000	0,0000000	0,0008728	0,0015306	0,0002119	0,0000000	0,0012209
alphaI cluster	0,0017880	0,0000000	0,0000000	0,0001965	0,0002095	0,0038983	0,0097113	0,0004767	0,0026119	0,0113063
Afipia	0,0146318	0,0000781	0,0384485	0,0000000	0,0000000	0,0000000	0,0000000	0,0000000	0,0000000	0,0000000
Rhodobacter	0,0015198	0,0000000	0,0000000	0,0001310	0,0006665	0,0090766	0,0089724	0,0015361	0,0025607	0,0133234
uncultured bacterium Clade III	0,0047382	0,0000000	0,0255759	0,0013097	0,1171329	0,0047710	0,0143558	0,0078394	0,0255045	0,0127926
Sphingobium	0,0010430	0,0000000	0,0008469	0,0009168	0,0000000	0,0000000	0,0000000	0,0000000	0,0000000	0,0000000
Sphingomonas	0,0008642	0,0000000	0,0154133	0,0007203	0,0000000	0,0000000	0,0000000	0,0000000	0,0000000	0,0000000
Sphingopyxis	0,0118604	0,0000000	0,0223577	0,0000000	0,0000000	0,0000000	0,0000000	0,0000000	0,0000000	0,0000000
Sphingorhabdus	0,0064666	0,0000000	0,0137195	0,0009168	0,0051035	0,0261244	0,0277089	0,0088988	0,0053262	0,0877435
Rheinheimera	0,0000596	0,0000000	0,0000000	0,0110667	0,0024565	0,0007564	0,0003167	0,0007945	0,0002561	0,0025479
Acidovorax	0,0351640	0,0000000	0,1212737	0,0011132	0,0002476	0,0026764	0,0030084	0,0047142	0,0103452	0,0055735
Duganella	0,0000596	0,0000000	0,0000000	0,0006548	0,0000000	0,0126258	0,1164300	0,0024366	0,0010243	0,0514889
Hydrogenophaga	0,2272253	0,0000000	0,0899390	0,0000000	0,0000000	0,0000000	0,0001056	0,0012183	0,0002049	0,0001592

Limnohabitans	0,0014900	0,0000000	0,0000000	0,0000000	0,1333955	0,1433060	0,0749459	0,0208697	0,0241729	0,0729869
Polaromonas	0,0004768	0,0000000	0,0082995	0,0000000	0,0190048	0,0008146	0,0017417	0,0012183	0,0021510	0,0019640
Polynucleobacter	0,0001788	0,0000000	0,0000000	0,0032742	0,0565765	0,0083202	0,0193698	0,0128714	0,0269384	0,0259568
Rhizobacter	0,0100128	0,0000000	0,0003388	0,0000000	0,0000000	0,0000582	0,0000528	0,0001589	0,0018949	0,0000000
Rhodoferax	0,0009536	0,0000000	0,0023713	0,0001310	0,0073125	0,0903590	0,0860822	0,4204672	0,3134282	0,0590796
Undibacterium	0,0000596	0,0000000	0,0000000	0,0000000	0,0018472	0,0080875	0,0358368	0,0010594	0,0005634	0,0076437
Variovorax	0,1758202	0,0000000	0,0008469	0,0001310	0,0001523	0,0001164	0,0000000	0,0001059	0,0000512	0,0001062
uncultured	0,0178502	0,0000000	0,0084688	0,0003274	0,0030850	0,0063420	0,0151475	0,0069919	0,0142886	0,0179946
Methylotenera	0,0021754	0,0000000	0,0074526	0,0000000	0,0007998	0,0762204	0,0059640	0,0424281	0,0490116	0,0064228
Legionella	0,0074798	0,0000781	0,0299797	0,0000000	0,0000000	0,0005237	0,0005806	0,0005827	0,0002561	0,0003185
Pseudomonas	0,0163900	0,0282157	0,1524390	0,0311047	0,0008760	0,0982719	0,1876286	0,0357540	0,0230462	0,0179946
Terrimicrobium	0,0000894	0,0000000	0,0000000	0,0259970	0,0024756	0,0000000	0,0014778	0,0001059	0,0002049	0,0017517
Opitutus	0,0004470	0,0000000	0,0177846	0,0000000	0,0018662	0,0000582	0,0003695	0,0000530	0,0001536	0,0001592
Bacillus	0,0000000	0,0339916	0,0000000	0,0000000	0,0000381	0,0000000	0,0000000	0,0000000	0,0000000	0,0000000
Paenibacillus	0,0000000	0,0801983	0,0000000	0,0000000	0,0000000	0,0000000	0,0000000	0,0000000	0,0000000	0,0001062
Clostridium sensu stricto 18	0,0000000	0,4066890	0,0000000	0,0000000	0,0000000	0,0000000	0,0000000	0,0000000	0,0000000	0,0000000
Shewanella	0,0000000	0,4090306	0,0000000	0,0172222	0,0000000	0,0000000	0,0000000	0,0000000	0,0000000	0,0000000
Serratia	0,0000000	0,0334452	0,0000000	0,0000000	0,0000000	0,0000000	0,0000000	0,0000000	0,0000000	0,0000000
uncultured bacterium Microtrichales	0,0000000	0,0000000	0,0003388	0,0199725	0,0011807	0,0000000	0,0000000	0,0000530	0,0000512	0,0000000
uncultured bacterium NS11-12 marine group	0,0000000	0,0000000	0,0003388	0,0000000	0,0140727	0,0000582	0,0006333	0,0010594	0,0014340	0,0002654
Pseudorhodobacter	0,0000000	0,0000000	0,0042344	0,0036671	0,0003428	0,0001746	0,0000528	0,0232534	0,0073236	0,0004777
Bacteriovorax	0,0000000	0,0000000	0,0309959	0,0000000	0,0000381	0,0000000	0,0000000	0,0000000	0,0000000	0,0000000
Candidatus Planktophila	0,0000000	0,0000000	0,0000000	0,0030122	0,0111401	0,0026183	0,0057001	0,0069919	0,0116255	0,0089708
Armatimonas	0,0000000	0,0000000	0,0000000	0,0163709	0,0008569	0,0000000	0,0000000	0,0000000	0,0000000	0,0000000
Pseudarcicella	0,0000000	0,0000000	0,0000000	0,0004584	0,0400472	0,0006400	0,0010556	0,0106997	0,0052750	0,0014332
GKS98 freshwater group	0,0000000	0,0000000	0,0000000	0,0007203	0,0169101	0,0000000	0,0001056	0,0016950	0,0027143	0,0004777
Acinetobacter	0,0000000	0,0000000	0,0000000	0,0165019	0,0000000	0,0642346	0,0000528	0,0182213	0,0006658	0,0001062
Arcobacter	0,0000000	0,0000000	0,0000000	0,0000000	0,0001143	0,0119858	0,0000528	0,0025425	0,0032265	0,0256383
Dechloromonas	0,0000000	0,0000000	0,0000000	0,0000000	0,0000381	0,0107639	0,0008445	0,0017480	0,0004609	0,0023887
Methylobacter	0,0000000	0,0000000	0,0000000	0,0000000	0,0000571	0,0079711	0,0051723	0,0143016	0,0134180	0,0049896
Others/Unclassified	0,2913252	0,0076881	0,2791328	0,1845328	0,2173938	0,3472974	0,2492743	0,2153716	0,2415241	0,3636605

Table S2. Metabolic reconstruction of Lake Pertusillo microbiomes. Metagenome prediction for Lake Pertusillo microbiomes was carried out in PICRUSt v2.0.0 and collapsed into MetaCyc metabolic pathways using the MinPath implementation of PICRUSt 2.0.0. Normalized abundances for MetaCyc pathways detected in each Lake Pertusillo sample is given below along with corresponding Metacyc pathway indices and MetaCyc pathway descriptions.

#MetaCyc pathway indices	Normalized abundance					MetaCyc pathway description
	1B	1B_LB	2B	M16	MM	
14DICHLORBENZDEGPWY	40,7257	0,12543	110,44	0,92824	8,87519	Degradation/Utilization/Assimilation. Aromatic Compounds Degradation. Chloroaromatic Compounds Degradation. Chlorobenzene Degradation.1,4dichlorobenzene degradation
PWY-6178	40,7257	0,12543	110,44	0,92824	8,87519	Degradation/Utilization/Assimilation. Aromatic Compounds Degradation. Chloroaromatic Compounds Degradation. Chlorotoluene Degradation.2,4,6trichlorophenol degradation
PWY-5642	1202,68	1342,19	1386,13	1151,62	1569,58	Degradation/Utilization/Assimilation. Aromatic Compounds Degradation. Nitroaromatic Compounds Degradation.Dinitrotoluene Degradation.2,4dinitrotoluene degradation
PWY-6210	221,927	0,68988	147,566	69,2251	9,53752	Degradation/Utilization/Assimilation.Aromatic Compounds Degradation. 2aminophenol degradation
PWY-5647	175,003	0,54375	159,942	99,5356	23,7113	Degradation/Utilization/Assimilation. Aromatic Compounds Degradation.Nitroaromatic Compounds Degradation. Nitrobenzoate Degradation.2nitrobenzoate degradation II
PWY-5648	611,645	10,3482	307,116	183,934	16,9556	Degradation/Utilization/Assimilation. Aromatic Compounds Degradation. Nitroaromatic Compounds Degradation. Nitrobenzoate Degradation.2nitrobenzoate degradation I
PWY-5641	515,595	34,7448	508,727	172,152	279,767	Degradation/Utilization/Assimilation. Aromatic Compounds Degradation. Nitroaromatic Compounds Degradation. Nitrotoluene Degradation.2-nitrotoluene degradation
PWY-5130	1701,36	23,1736	1459,8	2104,06	2522,14	Degradation/Utilization/Assimilation. Carboxylates Degradation. 2-Oxobutanoate Degradation.2-oxobutanoate degradation I
PWY-6094	40,7257	0,12543	110,44	0,92824	8,87519	Degradation/Utilization/Assimilation. Aromatic Compounds Degradation. Chloroaromatic Compounds Degradation. Chlorocatechol Degradation.3,4,6trichlorocatechol degradation

PWY-6217	1225,44	21,888	777,072	605,961	82,791	Degradation/Utilization/Assimilation.Aromatic Compounds Degradation.Chloroaromatic Compounds Degradation. Chlorobenzoate Degradation.3,4-dichlorobenzoate degradation
PWY-6084	40,7257	0,12543	110,44	0,92824	8,87519	Degradation/Utilization/Assimilation.Aromatic Compounds Degradation. Chloroaromatic Compounds Degradation.Chlorocatechol Degradation.3,5dichlorocatechol degradation
PWY-6088	725,961	45,532	680,261	439,235	183,995	Degradation/Utilization/Assimilation.Aromatic Compounds Degradation.Chloroaromatic Compounds Degradation. Chlorobenzoate Degradation.3-Chlorobenzoate Degradation. 3-chlorobenzoate degradation I (via chlorocatechol)
PWY-6216	1225,44	21,888	777,072	605,961	82,791	Degradation/Utilization/Assimilation. Aromatic Compounds Degradation. Chloroaromatic Compounds Degradation. Chlorobenzoate Degradation.3Chlorobenzoate Degradation.3-chlorobenzoate degradation II (via protocatechuate)
PWY-6089	2508,41	1280,85	1941,62	1631,81	955,474	Degradation/Utilization/Assimilation.Aromatic Compounds Degradation. Chloroaromatic Compounds Degradation. Chlorocatechol Degradation.3-Chlorocatechol Degradation.3-chlorocatechol degradation I (ortho)
PWY-6193	1326,06	640,489	1026,03	816,617	487,672	Degradation/Utilization/Assimilation. Aromatic Compounds Degradation.Chloroaromatic Compounds Degradation. Chlorocatechol Degradation.3-Chlorocatechol Degradation.3-chlorocatechol degradation II (ortho)
PWY-6214	424,399	0,7526	257,771	106,176	16,1608	Degradation/Utilization/Assimilation. Aromatic Compounds Degradation. Chloroaromatic Compounds Degradation. Chlorocatechol Degradation.3Chlorocatechol Degradation.3-chlorocatechol degradation III (meta pathway)
PWY-6104	899,351	1004,93	976,215	652,855	357,657	Degradation/Utilization/Assimilation. Aromatic Compounds Degradation.Chloroaromatic Compounds Degradation.Chlorotoluene Degradation.3 Chlorotoluene Degradation. 3-chlorotoluene degradation II
PWY0-1277	681,544	861,36	755,982	408,068	298,147	Degradation/Utilization/Assimilation. Aromatic Compounds Degradation.3phenylpropanoate and 3-(3-hydroxyphenyl)propanoate degradation

HCAMHPDEG-PWY	767,887	0	812,553	533,201	305,995	Degradation/Utilization/Assimilation.Aromatic Compounds Degradation. Phenolic Compounds Degradation .3-phenylpropanoate and 3-(3-hydroxyphenyl)propanoate degradation to 2hydroxypentadienoate
P281-PWY	840,905	316,748	544,444	465,315	84,4468	Degradation/Utilization/Assimilation. Aromatic Compounds Degradation.Phenolic Compounds Degradation.3-phenylpropanoate degradation
PWY-6093	40,7257	0,12543	110,44	0,92824	8,87519	Degradation/Utilization/Assimilation.Aromatic Compounds Degradation. Chloroaromatic Compounds Degradation.Chlorocatechol Degradation.4,5dichlorocatechol degradation
PWY-6535	654,386	707,564	518,126	530,309	1685,36	Degradation/Utilization/Assimilation.Amines and Polyamines Degradation.4Aminobutanoate Degradation.4-aminobutanoate degradation I
PWY-6537	1084,3	1330,02	990,432	757,942	1685,36	Degradation/Utilization/Assimilation.Amines and Polyamines Degradation.4Aminobutanoate Degradation.4-aminobutanoate degradation II
PWY-6536	2387,03	1367,21	2115,98	1791,57	2301,59	Degradation/Utilization/Assimilation.Amines and Polyamines Degradation.4Aminobutanoate Degradation.4-aminobutanoate degradation III
PWY-5022	230,853	504,85	256,361	151,865	500,455	Degradation/Utilization/Assimilation.Amines and Polyamines Degradation.4Aminobutanoate Degradation.4-aminobutanoate degradation V
PWY-6215	620,893	25,4001	495,333	356,533	97,1635	Degradation/Utilization/Assimilation.Aromatic Compounds Degradation.Chloroaromatic Compounds Degradation.Chlorobenzoate Degradation.4-chlorobenzoate degradation
PWY-6087	1274,57	640,489	1026,03	816,367	482,175	Degradation/Utilization/Assimilation.Aromatic Compounds Degradation.Chloroaromatic Compounds Degradation.Chlorocatechol Degradation.4-chlorocatechol degradation
PWY-6185	640,227	20,7384	538,334	209,436	183,641	Degradation/Utilization/Assimilation.Aromatic Compounds Degradation.4methylcatechol degradation (ortho cleavage)
PWY-5488	423,788	0,12543	253,306	11,3888	11,0609	Degradation/Utilization/Assimilation.Aromatic Compounds Degradation.Nitroaromatic Compounds Degradation.Nitrophenol Degradation.4Nitrophenol Degradation.4-nitrophenol degradation II
PWY-6041	40,7257	0,12543	110,44	0,92824	8,87519	Degradation/Utilization/Assimilation.Aromatic Compounds Degradation.Sulfoaromatic Compounds Degradation.4-sulfocatechol degradation

4TOLCARBDEG-PWY	2049,96	1053,04	1750,7	1417,12	1074,63	Degradation/Utilization/Assimilation.Aromatic Compounds Degradation .4-toluenecarboxylate degradation
TOLSULFDEG-PWY	2049,96	1053,04	1750,7	1417,12	1074,63	Degradation/Utilization/Assimilation.Aromatic Compounds Degradation.Sulfoaromatic Compounds Degradation.4-Toluenesulfonate Degradation.4-toluenesulfonate degradation I
PWY-5165	38,0173	21,888	214,77	45,0195	82,791	Degradation/Utilization/Assimilation.Aromatic Compounds Degradation.Sulfoaromatic Compounds Degradation.4-Toluenesulfonate Degradation..4-toluenesulfonate degradation II
AMMASSIM-PWY	2915,83	2646,12	2898,69	3046,65	4581,59	Degradation/Utilization/Assimilation.Inorganic Nutrients Metabolism.Nitrogen Compounds Metabolism.Ammonia Assimilation.ammonia assimilation cycle III
AMMOXID-PWY	0,29727	0	0,46996	1,82077	1,19219	Degradation/Utilization/Assimilation.Inorganic Nutrients Metabolism.Nitrogen Compounds Metabolism.Ammonia Oxidation.ammonia oxidation I (aerobic)
P303-PWY	151,111	11,2262	203,726	79,0073	44,9058	Degradation/Utilization/Assimilation.Inorganic Nutrients Metabolism.Nitrogen Compounds Metabolism.Ammonia Oxidation.ammonia oxidation II (anaerobic)
BENZCOA-PWY	1210,06	975,544	1125,88	912,743	624,221	Degradation/Utilization/Assimilation.Aromatic Compounds Degradation.anaerobic aromatic compound degradation (Thauera aromatica)
PWY-6533	38,0173	21,888	214,77	45,0195	82,791	Degradation/Utilization/Assimilation.Aromatic Compounds Degradation.aniline degradation
PWY-6079	611,645	10,3482	307,116	183,934	16,9556	Degradation/Utilization/Assimilation.Aromatic Compounds Degradation.2Aminobenzoate Degradation.anthranilate degradation I (aerobic)
PWY-6077	594,337	1,81877	345,652	367,725	38,1501	Degradation/Utilization/Assimilation.Aromatic Compounds Degradation.2Aminobenzoate Degradation.anthranilate degradation II (aerobic)
2AMINOBENZDEGPWY	578,516	1,12889	321,92	363,262	30,4671	Degradation/Utilization/Assimilation.Aromatic Compounds Degradation.2Aminobenzoate Degradation.anthranilate degradation III (anaerobic)
PWY-5431	945,206	132,695	843,1	314,994	275,025	Degradation/Utilization/Assimilation.Aromatic Compounds Degradation.Catechol Degradation.aromatic compounds degradation via β-ketoadipate
DESULFONATION-PWY	38,0173	21,888	214,77	45,0195	82,791	Degradation/Utilization/Assimilation.Aromatic Compounds Degradation.Sulfoaromatic Compounds Degradation.benzenesulfonate degradation

PWY-2503	28,5047	22,0761	172,474	43,6629	81,5988	Degradation/Utilization/Assimilation.Aromatic Compounds Degradation.Benzoate Degradation.benzoate degradation I (aerobic)
PWY-283	539,97	0,12543	323,329	277,686	1,32466	Degradation/Utilization/Assimilation. Aromatic Compounds Degradation.Benzoate Degradation.benzoate degradation II (aerobic and anaerobic)
PWY-5415	378,092	478,964	394,763	72,9498	215,786	Degradation/Utilization/Assimilation. Aromatic Compounds Degradation.Catechol Degradation.catechol degradation I (meta-cleavage pathway)
PWY-5420	422,572	478,964	394,763	103,665	215,786	Degradation/Utilization/Assimilation. Aromatic Compounds Degradation.Catechol Degradation.catechol degradation II (meta-cleavage pathway)
PWY-5417	683,936	22,8287	582,353	215,03	198,477	Degradation/Utilization/Assimilation. Aromatic Compounds Degradation.Catechol Degradation.catechol degradation III (ortho-cleavage pathway)
P183-PWY	515,595	34,7448	508,727	172,152	279,767	Degradation/Utilization/Assimilation. Aromatic Compounds Degradation.Catechol Degradation.catechol degradation to 2-hydroxypentadienoate I
PWY-5419	469,997	17,7487	383,249	139,164	147,964	Degradation/Utilization/Assimilation. Aromatic Compounds Degradation.Catechol Degradation.catechol degradation to 2-hydroxypentadienoate II
CATECHOL-ORTHOCLEAVAGE-PWY	482,763	19,003	518,244	99,2322	197,97	Degradation/Utilization/Assimilation. Aromatic Compounds Degradation.Catechol Degradation.catechol degradation to β-ketoadipate

PWY-6197	1381,54	1997,76	1668,11	1134,95	641,001	Degradation/Utilization/Assimilation. Aromatic Compounds Degradation.Chloroaromatic Compounds Degradation.chlorinated phenols degradation
PWY-6107	669,513	3,51211	578,985	442,769	27,1554	Degradation/Utilization/Assimilation. Aromatic Compounds Degradation H55:H92→ Chloroaromatic Compounds Degradation. chlorosalicylate degradation
PWY-481	3015,62	776,05	2510,97	2076,29	1489,31	Degradation/Utilization/Assimilation. Aromatic Compounds Degradation .ethylbenzene degradation (anaerobic)
P3-PWY	1757,73	1995,76	1839,48	1072,69	1404,66	Degradation/Utilization/Assimilation.Aromatic Compounds Degradation.Gallate Degradation.gallate degradation III (anaerobic)
PWY-6223	1059,1	625,093	686,37	550,873	136,837	Degradation/Utilization/Assimilation.Aromatic Compounds Degradation.Gentisate Degradation.gentisate degradation I
PWY-5923	109,362	0,06272	101,628	26,5976	244,465	Degradation/Utilization/Assimilation.Secondary Metabolites Degradation.Terpenoids Degradation.Limonene Degradation .limonene degradation I (D-limonene)

PWY-5924	109,362	0	101,628	26,5976	244,465	Degradation/Utilization/Assimilation.Secondary Metabolites Degradation.Terpenoids Degradation.Limonene Degradation .limonene degradation II (L-limonene)
PWY-5430	368,203	294,089	327,935	74,7803	171,755	Degradation/Utilization/Assimilation.Aromatic Compounds Degradation.Benzoate Degradation.meta cleavage pathway of aromatic compounds
PWY-6184	1298,3	6,89878	1047,53	884,61	45,4357	Degradation/Utilization/Assimilation.Aromatic Compounds Degradation .methylsalicylate degradation
PWY-5428	10,008	0,18815	44,1758	4,78399	74,3132	Degradation/Utilization/Assimilation.Aromatic Compounds Degradation.Xylene Degradation.m-xylene degradation to m-toluate
PWY-5427	0,13212	0	0	0,04748	0,22122	Degradation/Utilization/Assimilation.Aromatic Compounds Degradation.Naphthalene Degradation.naphthalene degradation (aerobic)
DENITRIFICATIONPWY	1172,03	678,119	889,626	555,122	332,29	Degradation/Utilization/Assimilation.Inorganic Nutrients Metabolism.Nitrogen Compounds Metabolism. Nitrate Reduction.nitrate reduction I (denitrification)
PWY0-1321	0	0,43901	0	0,0357	0,52986	Degradation/Utilization/Assimilation. Inorganic Nutrients Metabolism.Nitrogen Compounds Metabolism.Nitrate Reduction.nitrate reduction III (dissimilatory)
PWY-5674	0,03303	1235,01	0,70493	7,24739	78,1547	Degradation/Utilization/Assimilation.Inorganic Nutrients Metabolism.Nitrogen Compounds Metabolism.Nitrate Reduction.nitrate reduction IV (dissimilatory)
PWY-5675	1690,2	1280,7	1651,42	1441,96	2415,91	Degradation/Utilization/Assimilation.Inorganic Nutrients Metabolism.Nitrogen Compounds Metabolism.Nitrate Reduction.nitrate reduction V (assimilatory)
PWY490-3	1690,2	1280,7	1651,42	1441,96	2415,91	Degradation/Utilization/Assimilation.Inorganic Nutrients Metabolism.Nitrogen Compounds Metabolism.Nitrate Reduction.nitrate reduction VI (assimilatory)
PWY0-1352	57,9342	7469,69	131,353	493,573	929,378	Degradation/Utilization/Assimilation.Inorganic Nutrients Metabolism.Nitrogen Compounds Metabolism.Nitrate Reduction.nitrate reduction VIII (dissimilatory)
N2FIX-PWY	30,1562	624,528	55,4548	34,3805	54,8407	Degradation/Utilization/Assimilation.Inorganic Nutrients Metabolism.Nitrogen Compounds Metabolism.Nitrogen fixation.nitrogen fixation I (ferredoxin)
P221-PWY	2537,45	934,242	2356,83	1647,59	1888,96	Degradation/Utilization/Assimilation.Degradation/Utilization/Assimilation - Other.octane oxidation
PWY0-1337	5808,61	4686,85	5862,93	4437,83	4019,14	Degradation/Utilization/Assimilation.Fatty Acids and Lipids Degradation.Fatty Acids Degradation.oleate β-oxidation

PCPDEG-PWY	40,7257	0,12543	110,44	0,92824	8,87519	Degradation/Utilization/Assimilation.Chlorinated Compounds Degradation.pentachlorophenol degradation
PWY-5418	97,2396	0	64,8539	4,24847	11,5245	Degradation/Utilization/Assimilation.Aromatic Compounds Degradation.Phenolic Compounds Degradation.Phenol Degradation .phenol degradation I (aerobic)
PHENOLDEG-PWY	102,623	0,12543	109,03	0	0	Degradation/Utilization/Assimilation.Aromatic Compounds Degradation.Phenolic Compounds Degradation.Phenol Degradation .phenol degradation II (anaerobic)
PWY-6534	121,087	18,8149	127,358	6,56907	110,344	Degradation/Utilization/Assimilation.Aromatic Compounds Degradation.Phenolic Compounds Degradation.Phenylethylamine Degradation .phenylethylamine degradation II
PWY5F9-3233	363,129	22,8287	341,54	221,724	0	Degradation/Utilization/Assimilation.Aromatic Compounds Degradation.phthalate degradation
P184-PWY	725,234	631,218	509,197	402,712	161,343	Degradation/Utilization/Assimilation.Aromatic Compounds Degradation.Protocatechuate Degradation.protocatechuate degradation I (metacleavage pathway)
PROTOCATECHUATE-ORTHO-CLEAVAGE-PWY	1258,62	292,54	1179,12	479,453	436,143	Degradation/Utilization/Assimilation.Aromatic Compounds Degradation.Protocatechuate Degradation.protocatechuate degradation II (orthocleavage pathway)
PWY-6336	424,399	0,7526	257,771	106,176	16,1608	Degradation/Utilization/Assimilation.Aromatic Compounds Degradation.Protocatechuate Degradation.protocatechuate degradation III (paracleavage pathway)
PWY-5429	10,008	0,18815	44,1758	4,78399	74,3132	Degradation/Utilization/Assimilation.Aromatic Compounds Degradation.Xylene Degradation.p-xylene degradation to p-toluate
PWY-6183	1298,3	6,89878	1047,53	884,61	45,4357	Degradation/Utilization/Assimilation.Aromatic Compounds Degradation.Salicylate Degradation .salicylate degradation I
PWY-5340	1827,93	1359,75	1823,66	2071,61	1294,59	Degradation/Utilization/Assimilation.Inorganic Nutrients Metabolism.Sulfur Compounds Metabolism .sulfate activation for sulfonation

SO4ASSIM-PWY	1561,3	1366,24	1566,83	1641,93	1129,27	Degradation/Utilization/Assimilation.Inorganic Nutrients Metabolism.Sulfur Compounds Metabolism.Sulfate Reduction.sulfate reduction I (assimilatory)
SULFMETII-PWY	971,702	680,283	996,541	1166,05	732,203	Degradation/Utilization/Assimilation.Inorganic Nutrients Metabolism.Sulfur Compounds Metabolism.Sulfate Reduction.sulfate reduction II (assimilatory)
DISSULFRED-PWY	914,198	680,189	0	1035,95	647,425	Degradation/Utilization/Assimilation.Inorganic Nutrients Metabolism.Sulfur Compounds Metabolism.Sulfate Reductionsulfate reduction IV (dissimilatory, to hydrogen sufide))
P224-PWY	914,198	680,189	0	1035,95	647,425	Degradation/Utilization/Assimilation.Inorganic Nutrients Metabolism.Sulfur Compounds Metabolism.Sulfate Reduction.sulfate reduction V (dissimilatory, to thiosulfate)
P222-PWY	157,156	37,7552	327,559	335,094	117,232	Degradation/Utilization/Assimilation.Inorganic Nutrients Metabolism.Sulfur Compounds Metabolism.Sulfide Oxidation.sulfide oxidation I (sulfide-quinone reductase)
PWY-5276	56,1176	0	33,3669	36,4869	1,72205	Degradation/Utilization/Assimilation.Inorganic Nutrients Metabolism.Sulfur Compounds Metabolism.Sulfite Oxidation.sulfite oxidation I
PWY-5279	0,46242	0,62716	0	0,28561	0,26493	Degradation/Utilization/Assimilation.Inorganic Nutrients Metabolism.Sulfur Compounds Metabolism.Sulfite Oxidation.sulfite oxidation II
PWY-5278	1827,93	1359,75	1823,66	2071,61	1294,59	Degradation/Utilization/Assimilation.Inorganic Nutrients Metabolism.Sulfur Compounds Metabolism.Sulfite Oxidation.sulfite oxidation III
4AMINOBUTMETABPWY	869,343	1018,79	754,279	644,126	1172,65	Superpathway.superpathway of 4-aminobutanoate degradation
PWY-5183	745,74	376,543	713,498	314,773	261,547	Superpathway.superpathway of aerobic toluene degradation
PWY-2504	809,669	164,611	690,98	463,012	401,106	Superpathway.superpathway of aromatic compound degradation via 3-oxoadipate
PWY-6071	737,72	48,8559	560,305	578,56	509,794	Superpathway.superpathway of phenylethylamine degradation
PWY-6182	837,527	19,1284	698,648	382,425	167,337	Superpathway.superpathway of salicylate degradation
SULFATE-CYS-PWY	1893,23	1750,16	1972,64	1888,56	1775,01	Superpathway.superpathway of sulfate assimilation and cysteine biosynthesis
PWY-5294	106,637	18,8776	180,463	185,79	59,477	Superpathway.superpathway of sulfide oxidation (Acidithiobacillus ferrooxidans)
PWY-5335	56,1176	0	33,3669	36,4869	1,72205	Superpathway.superpathway of sulfide oxidation (Starkeya novella)

PWY-5308	942,025	680,189	928,515	1054,05	648,154	Superpathway.superpathway of sulfur metabolism (Desulfocapsa sulfoexigens)
PWY-5304	37,2411	15,24	34,1893	80,6317	57,2251	Superpathway.superpathway of sulfur oxidation (Acidianus ambivalens)
PWY-5306	914,198	680,189	0	1035,95	647,425	Superpathway.superpathway of thiosulfate metabolism (Desulfovibrio sulfodismutans)
PWY-6338	630,365	473,476	454,33	373,937	125,942	Superpathway.superpathway of vanillin and vanillate degradation
THIOSULFOX-PWY	46,2417	630,486	116,784	221,456	39,0773	Degradation/Utilization/Assimilation.Inorganic Nutrients Metabolism.Sulfur Compounds Metabolism.Thiosulfate Oxidation.thiosulfate oxidation I (to tetrathionate)
PWY-5303	18,3645	29,8529	35,0117	124,777	112,728	Degradation/Utilization/Assimilation.Inorganic Nutrients Metabolism.Sulfur Compounds Metabolism.Thiosulfate Oxidation.thiosulfate oxidation II (via tetrathionate)
PWY-5180	741,617	1134,68	857,669	446,589	375,54	Degradation/Utilization/Assimilation.Aromatic Compounds Degradation.Toluenes Degradation.toluene degradation I (aerobic) (via o-cresol)
PWY-5182	666,739	1133,3	781,224	397,678	336,198	Degradation/Utilization/Assimilation.Aromatic Compounds Degradation.Toluenes Degradation.toluene degradation II (aerobic) (via 4-methylcatechol)
PWY-5181	1156,18	527,506	1093,12	575,971	381,262	Degradation/Utilization/Assimilation.Aromatic Compounds Degradation.Toluenes Degradation.toluene degradation III (aerobic) (via p-cresol)
PWY-5178	586,595	691,346	627,344	310,838	283,635	Degradation/Utilization/Assimilation.Aromatic Compounds Degradation.Toluenes Degradation.toluene degradation IV (aerobic) (via catechol)
TOLUENE-DEG-3-OHPWY	1381,54	1997,76	1668,11	1134,95	641,001	Degradation/Utilization/Assimilation.Aromatic Compounds Degradation.Toluenes Degradation.toluene degradation to 2-hydroxypentadienoate (via 4methylcatechol)
TOLUENE-DEG-DIOLPWY	263,71	0	258,006	98,7859	0	Degradation/Utilization/Assimilation.Aromatic Compounds Degradation. Toluenes Degradation. toluene degradation to 2-hydroxypentadienoate (via toluene-cis-diol)
TOLUENE-DEG-2-OHPWY	948,565	1016,25	1088,42	653,551	460,384	Degradation/Utilization/Assimilation.Aromatic Compounds Degradation.Toluenes Degradation. toluene degradation to 2-hydroxypentadienoate I (via o-cresol)
TOLUENE-DEG-4-OHPWY	962,834	1009,76	1082,19	737,164	362,36	Degradation/Utilization/Assimilation. Aromatic Compounds Degradation. Toluenes Degradationtoluene degradation to 4-methylphenol
TOLUENE-DEG-CATECHOL-PWY	899,351	1004,93	976,215	652,855	357,657	Degradation/Utilization/Assimilation. Aromatic Compounds Degradation. Toluenes Degradationtoluene degradation to benzoate

PWY-81	34,45	0	2,81973	0,17851	0,13247	Degradation/Utilization/Assimilation. Aromatic Compounds Degradation. Toluenes Degradation.toluene degradation to benzoyl-CoA (anaerobic)
PWY-5179	354,153	361,12	345,476	61,0673	194,294	Degradation/Utilization/Assimilation. Aromatic Compounds Degradation. Toluenes Degradation.toluene degradation V (aerobic) (via toluene-cis-diol)
PWY-5184	2740,2	1797,89	2623,06	1902,61	1427,23	Degradation/Utilization/Assimilation. Aromatic Compounds Degradation. Toluenes Degradation.toluene degradation VI (anaerobic)
ALKANEMONOX-PWY	518,534	66,4792	890,566	166,512	303,744	Degradation/Utilization/Assimilation. Inorganic Nutrients Metabolism. Sulfur Compounds Metabolism .two-component alkanesulfonate monooxygenase
